# *Candida duobushaemulonii*: an emerging rare pathogenic yeast isolated from recurrent vulvovaginal candidiasis in Brazil

**DOI:** 10.1590/0074-02760160166

**Published:** 2016-06

**Authors:** Humberto Fabio Boatto, Sarah Desirée Barbosa Cavalcanti, Gilda MB Del Negro, Manoel João BC Girão, Elaine Cristina Francisco, Kelly Ishida, Olga Fischman Gompertz

**Affiliations:** 1 Universidade Federal de São Paulo, Departamento de Ginecologia, São Paulo, SP, Brasil; 2 Universidade Mogi das Cruzes, Faculdade de Medicina, Departamento de Clínica Médica, Mogi das Cruzes, SP, Brasil; 3 Universidade de São Paulo, Hospital das Clínicas da Faculdade de Medicina, São Paulo, SP, Brasil; 4 Universidade Federal de São Paulo, Departamento de Infectologia, São Paulo, SP, Brasil; 5 Universidade de São Paulo, Instituto de Ciências Biomédicas, Departamento de Microbiologia, São Paulo, SP, Brasil; 6 Universidade Federal de São Paulo, Departamento de Microbiologia, Imunologia e Parasitologia, São Paulo, SP, Brasil

**Keywords:** Candida duobushaemulonii, recurrent vulvovaginal candidiasis, antifungal susceptibility testing

## Abstract

The aim of this study was to identify *Candida* species isolated from women diagnosed with recurrent vulvovaginal candidiasis (RVVC) and their partners; and to evaluate the fluconazole (FLZ) susceptibility of the isolates. In a period of six years, among 172 patients diagnosed with vulvovaginal candidiasis, 13 women that presented RVVC and their partners were selected for this investigation. The isolates were obtained using Chromagar Candida medium, the species identification was performed by phenotypic and molecular methods and FLZ susceptibility was evaluated by E-test. Among 26 strains we identified 14*Candida albicans*, six *Candida duobushaemulonii*, four *Candida glabrata*, and two*Candida tropicalis*. Agreement of the isolated species occurred in 100% of the couples. FLZ low susceptibility was observed for all isolates of *C. duobushaemulonii* (minimal inhibitory concentration values from 8-> 64 µg/mL), two *C. glabrata*isolates were FLZ-resistant and all *C. albicans* and *C. tropicalis* isolates were FLZ-susceptible. This report emphasises the importance of accurate identification of the fungal agents by a reliable molecular technique in RVVC episodes besides the lower antifungal susceptibility profile of this rare pathogen *C. duobushaemulonii* to FLZ.

Vulvovaginal candidiasis (VVC) is a very common infection that affects a great number of women at reproductive age and the frequent cause of taking gynecological medical consultation. The recurrent vulvovaginal candidiasis (RVVC) is a more severe condition that affects 5-8% of these patients; and the reasons are poorly understood (Sobel 2007, Giraldo et al. 2013). *Candida albicans* is the most common causal agent but non-*albicans* species have been identified (Richter et al. 2005, Sobel 2007). Among *C.*non-*albicans*, *Candida glabrata* and *Candida tropicalis* had been related with RVVC cases. Interestingly, *C. glabrata* isolates present lower azoles susceptibility than other species (Richter et al. 2005, Sobel 2007).

The VVC and RVVC therapeutics are performed by topic application of polyene and azole agents (Sobel et al. 2004, Sobel 2007). Oral fluconazole (FLZ) has also been frequently used being the first drug of choice for VVC treatment (Sobel et al. 2004), and the Public Health Service in Brazil furnishes it to the patients with VVC or RVVC. It is important to highlight the reliable identification of*C.* non-*albicans* because they had shown highest minimal inhibitory concentration (MIC) values to many antifungal agents (Richter et al. 2005, Sobel 2007, Oberoi et al. 2012, Ramos et al. 2015). The aim of this report was to identify species of *Candida* clinical isolates from women diagnosed with RVVC and their partners; and to evaluate the FLZ susceptibility.

From July 2005 to August 2011, 2,026 female patients ranging from 18-65 years old were evaluated at Gynecologic Services of three private and two public Services in São Paulo city, São Paulo state, Brazil. Out of 172 patients who presented VVC, 13 women with RVVC and their partners were selected for this study. Secretion of the ectocervice and vagina from the women and of the foreskin and glans from their respective partners were collected with moistened swabs in sterile saline solution. In this study women with diabetes mellitus, on steroid, antibiotics or hormone therapy, in use of intrauterine device using vaginal douches or spermicidal, carriers of immunodeficiency virus were excluded. All procedures were previously approved by the Research Ethic Committee of São Paulo Hospital, Federal University of São Paulo, São Paulo, Brazil (Protocol CEP 1719/05).

Samples were previously cultivated in CHROMagar *Candida* medium® (Becton-Dickinson, New Jersey, USA) at 37ºC for 48 h and isolated yeasts were transferred to Sabouraud dextrose agar (Difco, USA) for further procedures. Morphological, biochemical and physiological characterisation of the isolates were defined according to Kurtzman et al. (2011). In order to confirm phenotypic identification, the isolates were submitted to molecular analysis. The DNA extractions and amplifications by polymerase chain reaction (PCR) were performed using *Candida* species-specific primers (Table) following protocols reported elsewhere (Luo & Mitchell 2002, Taira et al. 2014). Samples that were not identified by PCR were submitted to sequencing of the amplified products obtained with primers VLG/LS and ITS1/ITS4 (White et al. 1990), and the sequence similarity searches were done by BLAST (http://www.ncbi.nlm.nih.gov/blast).

**TABLE:** Primers used in polymerase chain reaction and random amplified polymorphic DNA assays: sequences, hybridisation temperature (annealing) and molecular weight of amplified products

Primers	Sequences	Annealing	Molecular weight	Reference
ITS1/4	F: 5’TCCGTAGGTGAACCTGCGG 3’ R: 5’TCCTCCGCTTATTGATATGC 3’	47ºC	variable	14
*Candida albicans* 1/2	F: 5’TTTATCAACTTGTCACACCAGA-3’ R: 5’ATCCCGCCTTACCACTACCG-3’	55ºC	272pb	14
*Candida glabrata* 1/2	F: 5’TTATCACACGACTCGACACT-3’ R: 5’CCCACATACTGATATGGCCTACAA-3’	52ºC	423pb	14
*Candida tropicalis* 1/2	F: 5’CAATCCTACCGCCAGAGGTTAT-3’ R: 5’TGGCCACTAGCAAAATAAGCGT-3’	52ºC	357pb	14

F: forward; R: reverse.

Twenty-six yeasts were isolated from 13 samples of women diagnosed with RVVC and from 13 samples of their partners. *C. albicans* (14), *Candida duobushaemulonii* (6), *C. glabrata* (4), and *C. tropicalis* (2) isolates were identified by phenotypic characteristics and confirmed by molecular methods. The identification of *C. albicans, C. glabrata* and *C. tropicalis* were confirmed by PCR assays and the remaining isolates (n = 6) were accurately identified as *C. duobushaemulonii* by sequencing of the region ITS of rDNA, showing 100% of identity with a reference strain available in GenBank (NCBI Reference Sequence NR130694.1). The isolates of *C. duobushaemulonii* obtained from samples collected from the three couples were evaluated by random amplified polymorphic DNA (RAPD) assay employing the primer OPG10 and amplification parameters previously described (Rocha et al. 2008); the yeast isolates demonstrated highly similar band patterns between isolates obtained of each couple (Figure). Thus, the *C. duobushaemulonii* isolated from the women with RVVC and from their partners were possibly the same isolates.

**Figure:**
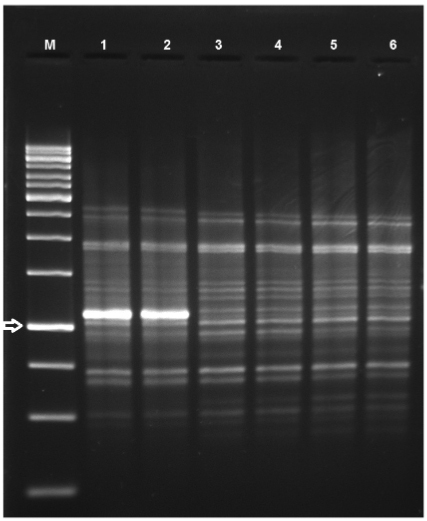
Random amplified polymorphic DNA banding pattern of *Candida duobushaemulonii* recovered from the three couples with the primer OPG 10. Lane M: molecular weight marker 1 Kb (arrow) (Fermentas, USA); Lines 1-2: *C. duobushaemulonii* isolated from the couple 1; Lines 3-4: *C. duobushaemulonii* isolated from the couple 2; and Lines 5-6: *C. duobushaemulonii* isolated from the couple 3.

*C. albicans*, *C. glabrata* and *C. tropicalis* have been commonly identified in RVVC (Richter et al. 2005, Sobel 2007). Here, we identified the three *Candida* species and also *C. duobushaemulonii* from genital samples of women and their partners. The infection of *Candida haemulonii* complex species (including *C. duobushaemulonii*) is not common in humans. Lehman et al. (1993) reported variations among *C. haemulonii* isolates and concluded that the isolates represented a complex of *C. haemulonii* group I and*C. haemulonii* group II species. In 2012, Cendejas-Bueno et al. (2012) proposed a reclassification of the isolates of group II as *C. duobushaemulonii.* Classical and commercial methods of yeast identification are not reliable to identify rare and emerging clinical isolates of *C. haemulonii* complex species as *C. haemulonii, Candida pseudohaemulonii*, *Candida auris* and *C. duoboshaemulonii* (Kim et al. 2009,Cendejas-Bueno et al. 2012). The identification at species level was achieved only after the sequencing of ITS region, which is in agreement with other authors (Cendejas-Bueno et al. 2012, Ramos et al. 2015).

The first human isolation of *C. haemulonii* complex species occurred in 1984 from the blood of a patient with renal failure (Lavarde et al. 1984). These species are opportunistic fungal pathogens that have been associated with onychomycosis, finger nail infections and broncho-alveolar lavage (Ramos et al. 2015), bloodstream infections (Ruan et al. 2010, Almeida Jr et al. 2012, Oberoi et al. 2012), fungaemia related catheter (Kim et al. 2011), and with an outbreak in neonatal care units (Khan et al. 2007). Recently, Almeida Jr et al. (2016) showed that among 14,642 positive yeast cultures from five hospitals in São Paulo (Brazil), 40 (0.3%) isolates were identified as *C. haemulonii* complex species. *C. duobushaemulonii* was characterised in nine biological samples and the data suggested that patients with diabetes mellitus are more likely to have positive cultures for *C. duobushaemulonii* (Almeida Jr et al. 2016).

In our study, the FLZ susceptibility testing of isolates was performed by the standard kit “E-Test” (Biodisk AB, Solna, Sweden) according to manufacturer recommendations. Reduced susceptibility to FLZ was observed for all isolates of *C. duobushaemulonii* (MIC ranging from 8->64 µg/mL), 50% of *C. glabrata* isolates exhibited resistance (≥ 64 µg/mL), and all isolates of*C. albicans* and *C. tropicalis* were susceptible to FLZ. The antifungal susceptibility interpretation was based on the breakpoint values for FLZ recommended by the CLSI (2012). The reduced susceptibility to FLZ of *C.* non-*albicans* species in RVVC cases described in this report, mainly *C. glabrata* and *C. duobushaemulonii*, is in agreement with other authors (Cendejas-Bueno et al. 2012, Almeida Jr et al. 2016). *C. duobushaemulonii* species complex have been isolated from several clinical sources varying from superficial to deep infections (Almeida Jr et al. 2012, Ramos et al. 2015) and they are related with lower susceptibility to polyenes, azoles and echinocandins (Ramos et al. 2015, Almeida Jr et al. 2016).

FLZ is the main antifungal agent employed in VVC; and for RVVC 10-14 days of induction therapy with a topical agent or oral FLZ, followed by FLZ, 150 mg weekly for six months, is strongly recommended (Sobel et al. 2004, Pappas et al. 2016). In our research FLZ was used for its effectiveness and because it is recommended and furnished by the Public System of Health in Brazil for RVVC treatment. In our experience, RVVC presented clinical and mycological resolution after symptomatic and asymptomatic partner’s treatment and the use of antifungal drugs for a long time.

The RVVC is a clinical condition that is characterised by three or more episodes of VVC with the isolation of the causal agent that occurs within a 12-months period. It is known that the causes of RVVC are multifactorial (Sobel 2007). The knowledge about the relation of sexual activity with the infection is limited. The role of sexual partners, trauma of the vaginal mucosa, immunosuppressive effect of semen during sexual activity besides other factors should be taken into consideration in the cases of repetition (Li et al. 2008). Studies that collect more specimens and detailed clinical data from women with RVVC may enhance the ability to distinguish between recurrence and reinfection. In all cases of RVVC of our investigation, there was 100% of agreement among *Candida* spp. isolated from the couples as demonstrated by the RAPD assay, that may suggest transmission between partners. Investigations did not prove the sexual acquisition of genital candidiasis (Sobel et al. 2004, Li et al. 2008, Giraldo et al. 2013). So, the real role of sexual transmission on RVVC has yet to be defined.

In summary, in these 13 cases of RVVC we emphasise the importance of the correct identification of emergent pathogens that had shown higher MIC values for routine antifungal drugs as FLZ. To our best knowledge, it is the first isolation of *C. duobushaemulonii*, a rare human emergent pathogen, from RVVC cases with the accurate identification of the fungal agent. We highlight the reduced susceptibility of this species to FLZ, which represents a major therapeutic choice for RVVC. All these informations will be very useful to improve the management of the patients with infections caused by these organisms and will contribute to the surveillance of RVVC.

## References

[B1] Almeida JN, Assy JGPL, Levin AS, Del Negro GMB, Giudice MC, Tringoni MP (2016). Candida haemulonii complex species, Brazil, January 2010 - March
2015. Emerg Infect Dis.

[B2] Almeida JN, Motta AL, Rossi F, Abdala E, Pierrotti LC, Kono ASG (2012). First report of clinical isolate of Candida haemulonii in
Brazil. Clinics.

[B3] Cendejas-Bueno E, Kolecka A, Alastruey-Izquierdo A, Theelen B, Groenewald M, Kostrzewa M (2012). Reclassification of the Candida haemulonii complex as Candida
haemulonii (C. haemulonii group I), C. duobushaemulonii sp. nov. (C. haemulonii
group II), and C. haemulonii var. vulnera var. nov.: three multiresistant human
pathogenic yeasts. J Clin Microbiol.

[B4] CLSI - Clinical Laboratory Standards Institute (2012). Reference method for broth dilution antifungal susceptibility testing of
yeasts; Fourth informational supplement. CLSI document M27-S4.

[B5] Giraldo PC, Rodrigues HM, Melo AG, do Amaral RL, Passos MRL, Eleutério J (2013). Vulvovaginitis and the treatment of asymptomatic partners: a
systematic review and metanalisis. DST-J Bras Doenças Sex Transm.

[B6] Khan ZU, Al-Sweih NA, Ahmad S, Al-Kazemi N, Khan S, Joseph L (2007). Outbreak of fungemia among neonates caused by Candida haemulonii
resistant to amphotericin b, itraconazole , and fluconazole. J Clin Microbiol.

[B7] Kim MN, Shin JH, Sung H, Lee K, Ec Kim, Ryoo N (2009). Candida haemulonii and closely related species at 5 university
hospitals in Korea; identification, antifungal susceptibility, and clinical
features. Clin Infect Dis.

[B8] Kim S, Ko KS, Moon SY, Lee MS, Lee MY, Son JS (2011). Catheter-related candidemia caused by Candida haemulonii in a patient
in long-term care hospital. J Korean Med Sci.

[B9] Kurtzman CP, Fell JW, Boekhout T, Robert V, Kurtzman CP, Fell JW, Boekhout T (2011). Methods for isolation, phenotypic characterization and
maintenance of yeasts. The Yeasts, a Taxonomic Study.

[B10] Lavarde V, Daniel F, Saez H, Arnold M, Faguer B (1984). Peritonite mycosique a Torulopsis haemulonii. Bul Soc Fr Mycol Med.

[B11] Lehmann PF, Wu LC, Pruitt WR, Meyer SA, Ahearn DG (1993). Unrelatedness of groups of yeasts within the Candida haemulonii
complex. J Clin Microbiol.

[B12] Li J, Fan RS, Liu XP, Li DM, Nie ZH, Li F (2008). Biased genotype distributions of Candida albicans strains associated
with vulvovaginal candidosis and candidal balanopostitis in China. Clin Infect Dis.

[B13] Luo G, Mitchell TG (2002). Rapid identification of pathogenic fungi directly from cultures by
using multiplex PCR. J Clin Microbiol.

[B14] Oberoi JK, Wattal C, Goel N (2012). Non-albicans Candida species in blood stream infections in a tertiary
care hospital at New Delhi, India. Indian J Med Res.

[B15] Pappas PG, Kauffman CA, Andes DR, Clancy CJ, Marr KA, Ostrosky-Zeichner L (2016). Clinical practice guideline for the management of candidiasis: 2016
Update by the Infectious Diseases Society of America. Clin Infect Dis.

[B16] Ramos LS, Figueiredo-Carvalho MH, Barbedo LS, Ziccardi M, Chaves AL, Zancopé-Oliveira RM (2015). Candida haemulonii complex: species identification and antifungal
susceptibility profiles of clinical isolates from Brazil. J Antimicrob Chemother.

[B17] Richter SS, Galask RP, Messer SA, Hollis RJ, Diekema DJ, Pfaller MA (2005). Antifungal susceptibilities of Candida species causing vulvovaginitis
and epidemiology of recurrent cases. J Clin Microbiol.

[B18] Rocha BA, Del Negro GMB, Yamamoto L, Souza MVB, Precioso AR, Okay TS (2008). Identification and differentiation of Candida species from pediatric
patients by random amplified polymorphic DNA. Rev Soc Bras Med Trop.

[B19] Ruan SY, Kuo YW, Huang CT, Hsiue HC, Hsueh PR (2010). Infections haemulonii due to Candida: species identification,
antifungal susceptibility and outcomes. Int J Antimicrob Agents.

[B20] Sobel JD, Wiesenfeld HD, Martens MGE, Danna P, Hooton TM, Rompalo A (2004). Maintenance fluconazole therapy for recurrent vulvovaginal
candidiasis. N Engl J Med.

[B21] Sobel JD (2007). Vulvovaginal candidosis. Lancet.

[B22] Taira CL, Okay TS, Delgado AF, Ceccon MEJR, Almeida MTG, Del Negro GMB (2014). A multiplex nested PCR for the detection and identification of Candida
species in blood samples of critically ill paediatric patients. BMC Infect Dis.

[B23] White TJ, Bruns T, Lee S, Taylor J, Inns MA, Gelfand DH, Sninsky J, White TJ (1990). Amplification and direct sequencing of fungal ribosomal
RNA genes for phylogenetic. PCR protocols a guide to methods and applications.

